# Effect of Surface Treatments and Repair Resins on the Flexural Strength of Heat-Polymerized Denture Base Resin: An In Vitro Study

**DOI:** 10.7759/cureus.80007

**Published:** 2025-03-04

**Authors:** Kimmi Gupta, Alisha Chuchra, Shweta Bindra, Reetu Arora, Nupur Hingad, Amit Babbar

**Affiliations:** 1 Department of Prosthodontics, Adesh Institute of Dental Sciences and Research, Bathinda, IND; 2 Department of Orthodontics, Adesh Institute of Dental Sciences and Research, Bathinda, IND; 3 Department of Prosthodontics, Sri Sukhmani Dental College and Hospital, Dera Bassi, IND; 4 Department of Conservative Dentistry and Endodontics, Adesh Institute of Dental Sciences and Research, Bathinda, IND; 5 Department of Oral and Maxillofacial Pathology and Microbiology, Sri Sukhmani Dental College and Hospital, Dera Bassi, IND

**Keywords:** alumina paticles, autopolymerizing resin, flexural strength, heat polymerizing resin, laser, light polymerizing resin, phosphoric acid

## Abstract

Objective: Our research compared the flexural strength of heat-polymerized denture base resin after applying different surface treatments and using various repair materials.

Methodology: A total of 96 rectangular specimens made of heat-polymerized acrylic resin were prepared using a customized metal mold following conventional processing methods. Each specimen measured 65 × 10 × 2.5 mm. The specimens were then sectioned into two halves, creating a 3 × 10 × 2.5 mm repair gap. Three groups were formed (n = 32 each) based on the type of repair material used and subdivided into four subgroups (n = 8 each) according to the surface treatment applied. These subgroups included a control group with no surface treatment, a group treated with 37% phosphoric acid, a group treated with 250 µm alumina particles, and a group treated with an Er: YAG laser. The designated repair material was used to repair the gap following surface treatment. The flexural strength of all specimens was then evaluated using a universal testing machine to evaluate the effects of these treatments and repair resins. The recorded flexural strength values were tabulated and analyzed using statistical methods.

Results: The repair material (group factor) had a significant impact on flexural strength (p < 0.001), with a sum of squares of 166,506.176, a mean square of 83,253.088, and an F-ratio of 24,560.779, demonstrating that the type of resin used for repair influenced the strength of the repaired specimen. The surface treatment (subgroup factor) also had a significant effect (p < 0.001), with a sum of squares of 4,230.983, a mean square of 1,410.328, and an F-ratio of 416.066, confirming that surface treatments improved the flexural strength of the repaired denture base. The interaction between repair material and surface treatment was also significant (p < 0.001), with a sum of squares of 2,357.973, a mean square of 392.996, and an F-ratio of 115.939, suggesting that selecting the appropriate combination of repair material and surface treatment is essential for achieving optimal mechanical strength.

Conclusion: This study's findings indicate that acrylic resin denture repairs can be effectively performed using heat-polymerized, auto-polymerized, or light-polymerized acrylic resin. The results also demonstrated that applying surface treatments to the repair interface enhances repair strength, thereby improving the durability and performance of the repaired denture base.

## Introduction

Tooth loss is a common issue affecting both function and appearance, often requiring dentures to restore chewing ability and aesthetics. Polymethylmethacrylate (PMMA) acrylic resin is the most widely used material for dentures due to its favorable appearance. However, despite its popularity, it lacks the required strength, making it prone to fractures over time [1]. Dentures can break both inside and outside the mouth. External fractures usually result from accidental drops, whereas internal fractures arise from excessive biting force, poor fit, high frenal attachment, improper occlusion, or material limitations. Midline fractures are the most common and result from continuous bending of the denture base during use. Studies have shown that lower dentures break more often than upper dentures, with a reported ratio of 2:1, according to studies [2-5].

Repairing broken dentures is necessary to restore their function and extend their lifespan. The primary goal of denture repair is to create a strong bond between the fractured parts while keeping the process quick and affordable. Studies indicate that in repaired dentures, fractures most commonly occur at the junction between the old and new materials rather than at the center of the repaired area. Good adhesion between the repair material and the denture base is essential for strengthening the repair and preventing further fractures [6,7].

Several materials are available for denture repair, including heat-, auto-, visible light-, and microwave-polymerized acrylic resins. The strength and durability of acrylic resin improve when polymerization occurs at higher temperatures. However, heat-polymerized resin requires complex procedures that take time and may cause slight changes in denture shape due to heat. Although auto- and light-polymerizing resins have lower strength, autopolymerized resin is widely used because it is quick and cost-effective. Light-polymerized resins are becoming more popular because they offer good strength, are easy to handle, require a short curing time, and can be applied directly in the mouth without flasking [8-10]. The choice of repair material depends on factors such as working time, strength, and dimensional stability [10].

Different surface treatments are used to improve the bond strength between the denture base and the repair material. These include both mechanical and chemical methods [11]. Mechanical surface treatments involve bur grinding, sandblasting with aluminum oxide, and laser application [12]. Chemical treatments use substances like chloroform and methylene chloride, but these chemicals have harmful effects. Safer alternatives include acetone, ethyl acetate, methyl methacrylate, methyl formate, and phosphoric acid [13,14]. Phosphoric acid treatment is especially effective as it increases the bonding ability of PMMA and creates a rough surface at a microscopic level, improving the attachment of the repair material [15].

Air abrasion is another standard method for surface treatment. It involves spraying aluminum oxide particles under high pressure to clean the surface, make it rougher, and increase the bonding area [16-18]. Lasers have been widely used in dentistry since the development of ruby lasers in 1960, and their applications continue to expand. The erbium-doped yttrium aluminum garnet (Er:YAG) laser, which works at a wavelength of 2.94 μm, is FDA-approved for use in complex tissues like teeth and bones. It is used in cavity preparation, caries removal, and root canal treatment. Recently, lasers have been used to modify denture surfaces, providing a safe and effective way to improve bonding [19,20].

Another important factor in denture repair is maintaining an optimal gap between the fractured parts. Different studies suggest various repair gaps, with some recommending a 1.5-3 mm gap and others suggesting up to 10 mm [19]. This technique ensures that any shrinkage occurs on the polished surface rather than the fitting surface, preserving the overall accuracy of the denture repair [11,20].

Although denture fractures are common, research on how different surface treatments affect the strength of repaired dentures remains limited. This study evaluates and compares the flexural strength of heat-polymerized denture base resin after applying different surface treatments and using various repair materials.

## Materials and methods

**Figure 1:**
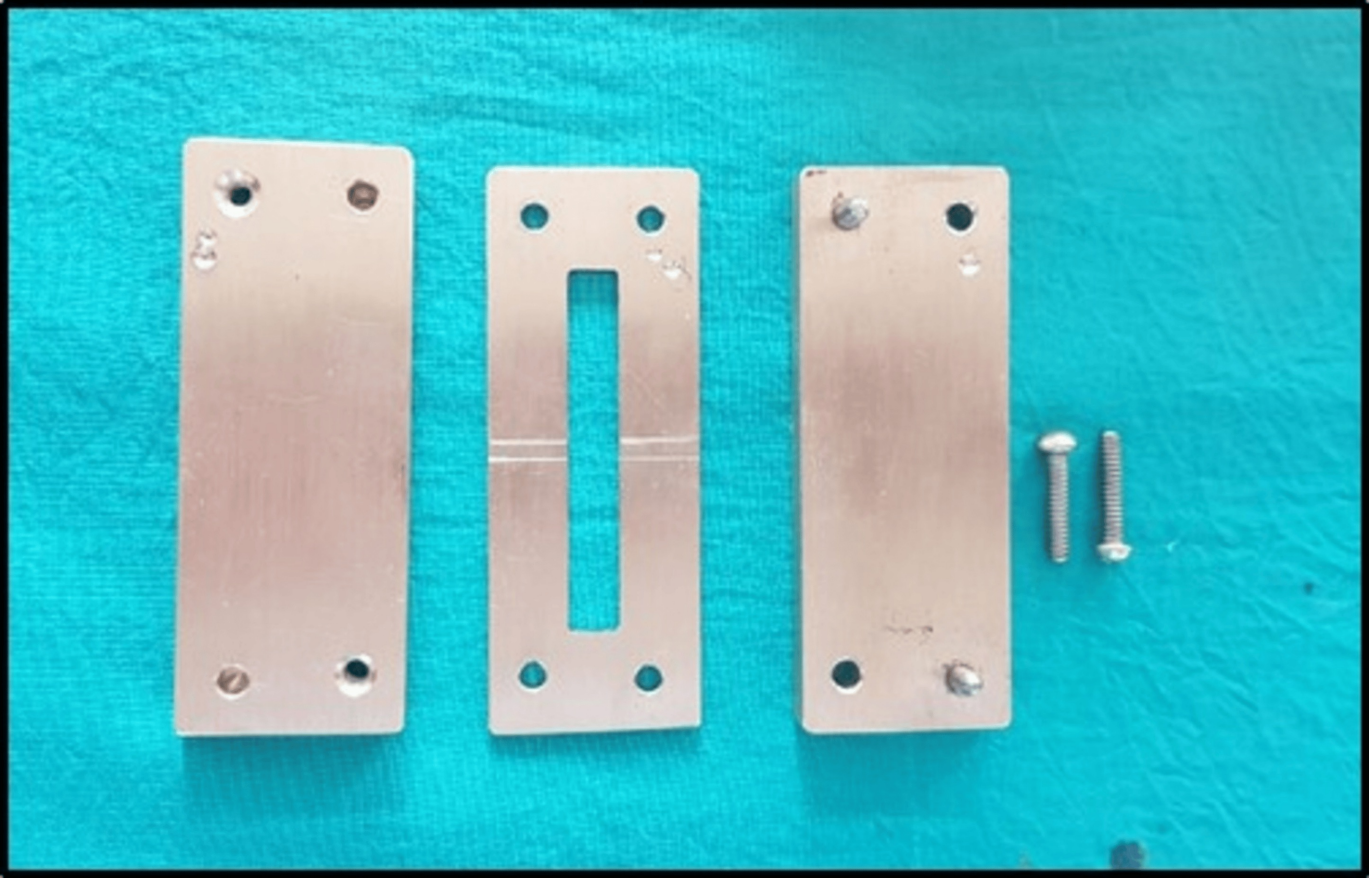
Metal mold

**Figure 2:**
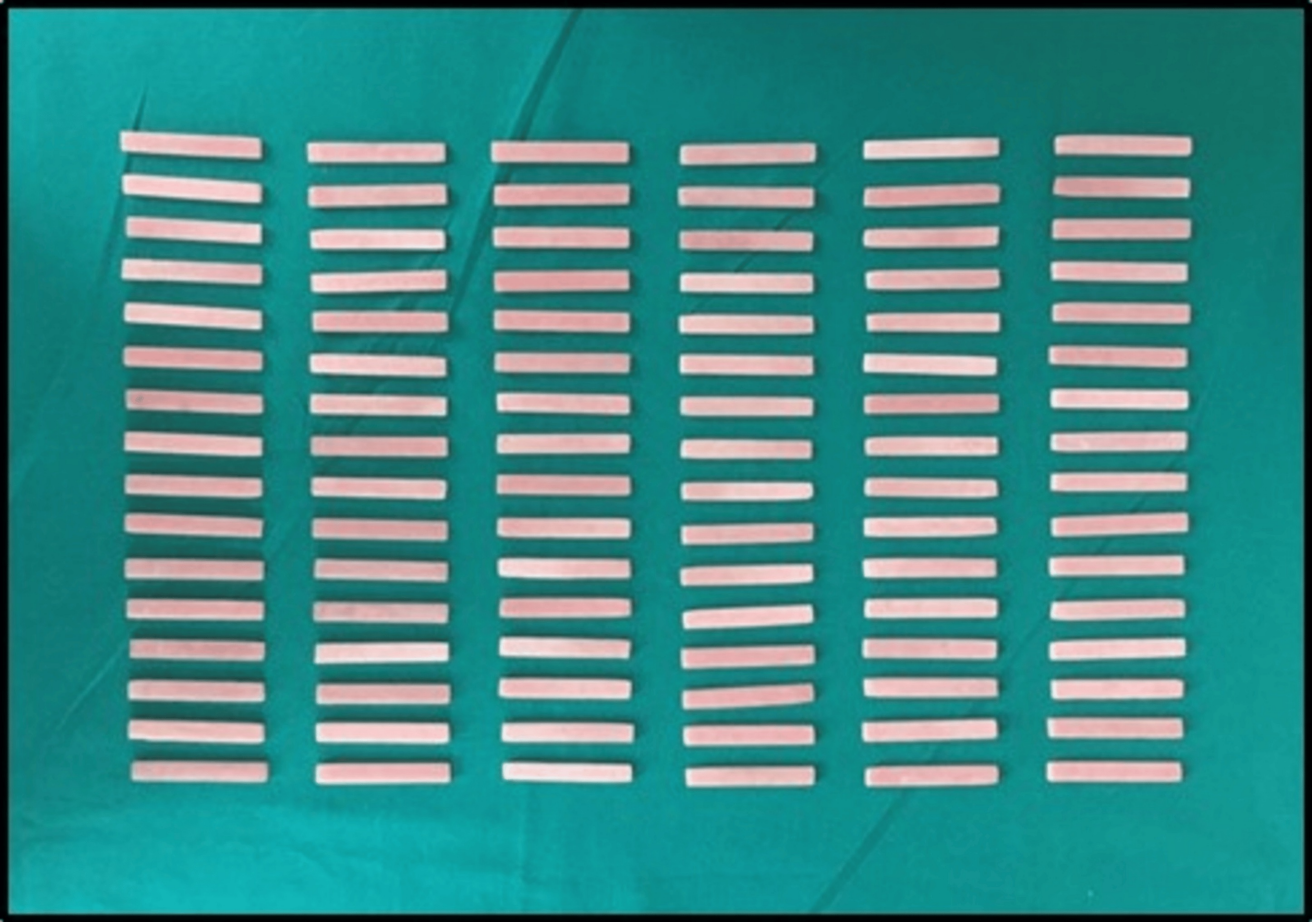
Heat-polymerized resin specimens

The strength of the repaired heat-polymerized denture base resin was tested in vitro using various repair materials and surface treatments. A power analysis estimated that 96 specimens were required for the study. Rectangular specimens measuring 65 × 10 × 2.5 mm were fabricated from heat-polymerized acrylic resin (Trevalon HI; Dentsply Sirona Inc., Charlotte, North Carolina, United States) using a customized brass mold and conventional processing methods to ensure consistency (Figure 1). Each specimen was partially cut using a carborundum disc to create a 3 × 10 × 2.5 mm repair gap (Figure 2).

After cutting, the specimens were randomly assigned to three groups (n = 32 per group) according to the repair material used. Group H was repaired with heat-polymerizing resin; Group A with autopolymerizing resin; and Group L with light-polymerizing resin. Each group was further divided into four subgroups (n = 8 per subgroup) based on the surface treatment applied. Subgroup C served as the control group and did not receive any treatment. Subgroup E_p _underwent phosphoric acid treatment; subgroup E_a_ was treated with alumina particles; and subgroup E_l_ received laser treatment.

Surface treatment procedures

Surface treatments were applied to enhance the bond strength between the denture base and the repair material. In Subgroup E_p_, specimens were etched with 37% phosphoric acid (DPI) for 30 seconds, followed by rinsing with a water spray for 30 seconds and air-drying for 20 seconds. In Subgroup E_a_, specimens were sandblasted with 250 µm aluminum oxide particles (Korox® 250; BEGO GmbH & Co. KG. Bremen, Germany) for 10 seconds at a pressure of 0.2 MPa, maintaining a 10 mm distance. In Subgroup E_l_, specimens were treated with an Er:YAG laser (wavelength: 2940 nm, spot size: 0.8 mm, pulse frequency: 10 Hz, pulse energy: 150 mJ, pulse duration: 100 ms). The laser was applied for 60 seconds with water irrigation, maintaining a 10 mm distance between the laser tip and each specimen.

Scanning electron microscopy (SEM) analysis

Two randomly selected specimens from each subgroup were examined using an SEM to evaluate the surface characteristics after treatment. The SEM provided high-resolution images of the treated surfaces at 2000× magnification, enabling a detailed analysis of surface morphology.

Repair process

After cutting, the specimens were repaired using their designated resins, with the repair process varying based on the material type. In Group H, heat-polymerizing resin was packed into the repair gap and polymerized following the manufacturer’s instructions. In Group A, autopolymerizing resin was prepared at a P/L ratio of 2:1, manually packed into the repair gap, and bench-polymerized for 15 minutes at room temperature. In Group L, light-polymerizing denture base material in premixed sheet form was adapted into the joint space and placed in a light-curing chamber for 10 minutes (five minutes per surface) to achieve complete polymerization. All repaired specimens were stored in distilled water at 37°C for seven days before mechanical testing.

Mechanical testing

The flexural strength of the repaired specimens was evaluated using a universal testing machine (UTM) with a crosshead speed of 5 mm/minute. The specimens were placed on two supporting rollers, ensuring that the center of each specimen was aligned with the center of the rollers’ span. A centrally located rod was used to apply force until the specimen fractured. The three-point bending test method was used to assess the strength of the repaired specimens.

Data analysis

The mean values and standard deviations were calculated for each group after recording test results. Differences among the groups were analyzed using a two-way ANOVA. For further comparisons, a post hoc Tukey HSD test was performed. A p-value less than 0.05 was considered statistically significant.

## Results

The overall model demonstrated a sum of squares of 465,154.095, with a mean square value of 38,762.841 and an F-ratio of 11,435.559, which was statistically significant (p < 0.001). These results confirm that the analyzed factors significantly influenced the findings. The repair material (group factor) significantly affected flexural strength (p < 0.001), with a sum of squares of 166,506.176, a mean square of 83,253.088, and an F-ratio of 24,560.779, demonstrating that the type of resin used for repair influenced the strength of the repaired specimen. The surface treatment (subgroup factor) also had a significant effect (p < 0.001), with a sum of squares of 4,230.983, a mean square of 1,410.328, and an F-ratio of 416.066, confirming that surface treatments improved the flexural strength of the repaired denture base. The interaction between repair material and surface treatment was significant (p < 0.001), with a sum of squares of 2,357.973, a mean square of 392.996, and an F-ratio of 115.939. This suggests that choosing the right repair material and surface treatment combination is essential for achieving optimal mechanical strength. The error term had a sum of squares of 284.733 and a mean square value of 3.390, reflecting minimal variability within the groups. Based on an analysis of 96 specimens, the total sum of squares for the study was 465,438.828. These findings demonstrate that both the choice of repair material and the application of surface treatments significantly enhance the flexural strength of denture base resin (Table 1).

**Table 1:** Two-way analysis of variance (ANOVA) to evaluate significant differences among groups R Squared = 0.999 (Adjusted R Squared = 0.999) a: computed using alpha = 0.05 **p<0.001; highly significant

Source	Type III Sum of Squares	Df	Mean Square	F ratio	P value
Model	465154.095^a^	12	38762.841	11435.559	<0.001**
Group	166506.176	2	83253.088	24560.779	<0.001**
Sub group	4230.983	3	1410.328	416.066	<0.001**
Group x Sub group	2357.973	6	392.996	115.939	<0.001**
Error	284.733	84	3.390	-	-
Total	465438.828	96	-	-	-

A post hoc Tukey's Honestly Significant Difference (HSD) test compared the flexural strength among different subgroups within each repair material group. The results revealed significant differences (p < 0.001) in most comparisons, confirming the substantial effect of surface treatments on flexural strength. In Group H (heat-polymerizing resin), the control subgroup (subgroup C) exhibited the lowest flexural strength (103.0863 ± 2.04767 MPa). The application of phosphoric acid (subgroup E_^p^_) resulted in a modest increase (106.8738 ± 1.71104 MPa, p < 0.001). Air abrasion (subgroup E_a_) further enhanced flexural strength (116.8312 ± 1.14405 MPa, p < 0.001), while laser treatment (subgroup E_l_) produced the highest flexural strength (125.1263 ± 0.88972 MPa, p < 0.001). Pairwise comparisons indicated that laser treatment was the most effective, followed by air abrasion and phosphoric acid. In Group A (autopolymerizing resin), the control subgroup had the lowest flexural strength (24.9225 ± 2.14143 MPa). Phosphoric acid treatment led to a moderate increase (29.7813 ± 1.95319 MPa, p = 0.002), whereas air abrasion resulted in a substantial improvement (52.7800 ± 2.83373 MPa, p < 0.001). Laser treatment also enhanced strength (36.2963 ± 1.63432 MPa, p < 0.001), but air abrasion yielded the highest improvement. 

Statistically significant differences among subgroups confirmed the positive effect of surface treatments on repaired specimens’ strength. In Group L (light-polymerizing resin), the control subgroup exhibited the lowest flexural strength (11.4425 ± 2.03958 MPa). Phosphoric acid treatment improved strength (18.8250 ± 1.17287 MPa, p < 0.001), while air abrasion resulted in a more significant increase (22.8488 ± 1.95952 MPa, p < 0.001). Laser treatment had a relatively minor effect (13.0688 ± 1.72574 MPa, p = 0.349) and was not significantly different from the control group. These findings indicate that air abrasion was the most effective surface treatment for improving flexural strength in specimens repaired with light-polymerizing resin. Overall, the results confirm that surface treatments significantly improved the flexural strength of all repair materials. Laser treatment was the most effective for heat-polymerizing resin, while air abrasion provided the most significant improvement for both autopolymerizing and light-polymerizing resin repairs. These findings suggest that choosing the best combination of repair material and surface treatment is essential for achieving stronger and more durable denture base repairs (Tables 2, 3).

**Table 2:** Average flexure strength among subgroups

Group	Sub-group	Total number	Flexure strength (MPa), mean ± SD
Heat-polymerizing resin (H)	Sub Group C	8	103.08 ± 2.04
	Sub Group E_p_	8	106.87 ± 1.71
	Sub Group E_a_	8	116.83 ± 1.14
	Sub Group E_I_	8	125.12 ± 0.88
Autopolymerizing resin (A)	Sub Group C	8	24.92 ± 2.14
	Sub Group E_p_	8	29.78 ± 1.95
	Sub Group E_a_	8	52.78 ± 2.83
	Sub Group E_I_	8	36.29 ± 1.63
Light-polymerizing resin (L)	Sub Group C	8	11.44 ± 2.03
	Sub Group E_p_	8	18.82 ± 1.17
	Sub Group E_a_	8	22.84 ± 1.95
	Sub Group E_I_	8	13.06 ± 1.72

**Table 3:** Multiple comparison of flexure strength among sub-groups using Post Hoc Tukey HSD *p<0.05: significant; **p<0.001: highly significant HSD: Honestly Significant Difference

Groups	Mean difference of flexure strength (MPa)	p-value
Heat-polymerizing resin (H) group		
Subgroup C vs E_p_	3.787	<0.001**
Subgroup C vs E_a_	13.74	<0.001**
Subgroup C vs E_I_	22.04	<0.001**
Subgroup E_p_vs E_a_	9.95	<0.001**
Subgroup E_p_vs E_I_	18.25	<0.001**
Subgroup E_a_ vs E_I_	8.29	<0.001**
Autopolymerizing resin (A)		
Subgroup C vs E_p_	4.85	0.002*
Subgroup C vs E_a_	27.85	<0.001**
Subgroup C vs E_I_	11.37	<0.001**
Subgroup E_p_vs E_a_	22.99	<0.001**
Subgroup E_p_vs E_I_	6.51	<0.001**
Subgroup E_a_ vs E_I_	16.48	<0.001**
Light-polymerizing resin (L)		
Subgroup C vs E_p_	7.38	<0.001**
Subgroup C vs E_a_	11.40	<0.001**
Subgroup C vs E_I_	1.62	0.349
Subgroup E_p_vs E_a_	4.02	0.001*
Subgroup E_p_vs E_I_	5.75	<0.001**
Subgroup E_a_ vs E_I_	9.78	<0.001**

**Figure 3:**
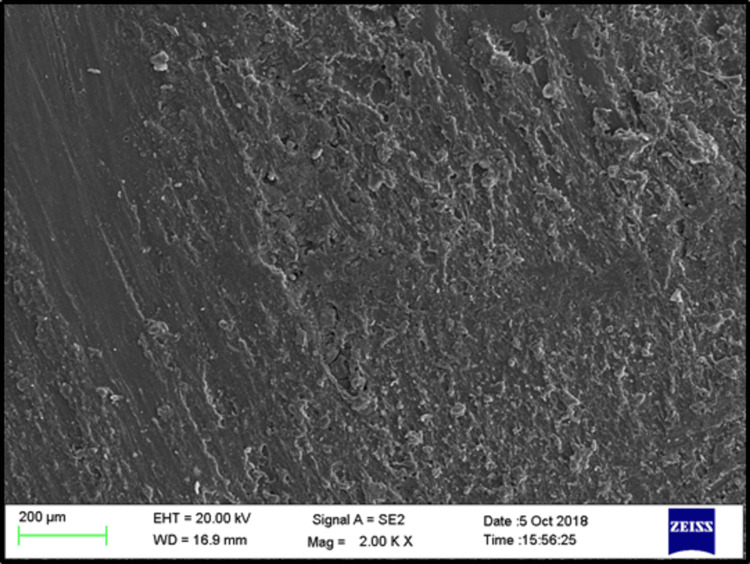
SEM evaluation for sub-group C (control) EHT: electron high tension; WD: working distance; SEM: scanning electron microscope

**Figure 4:**
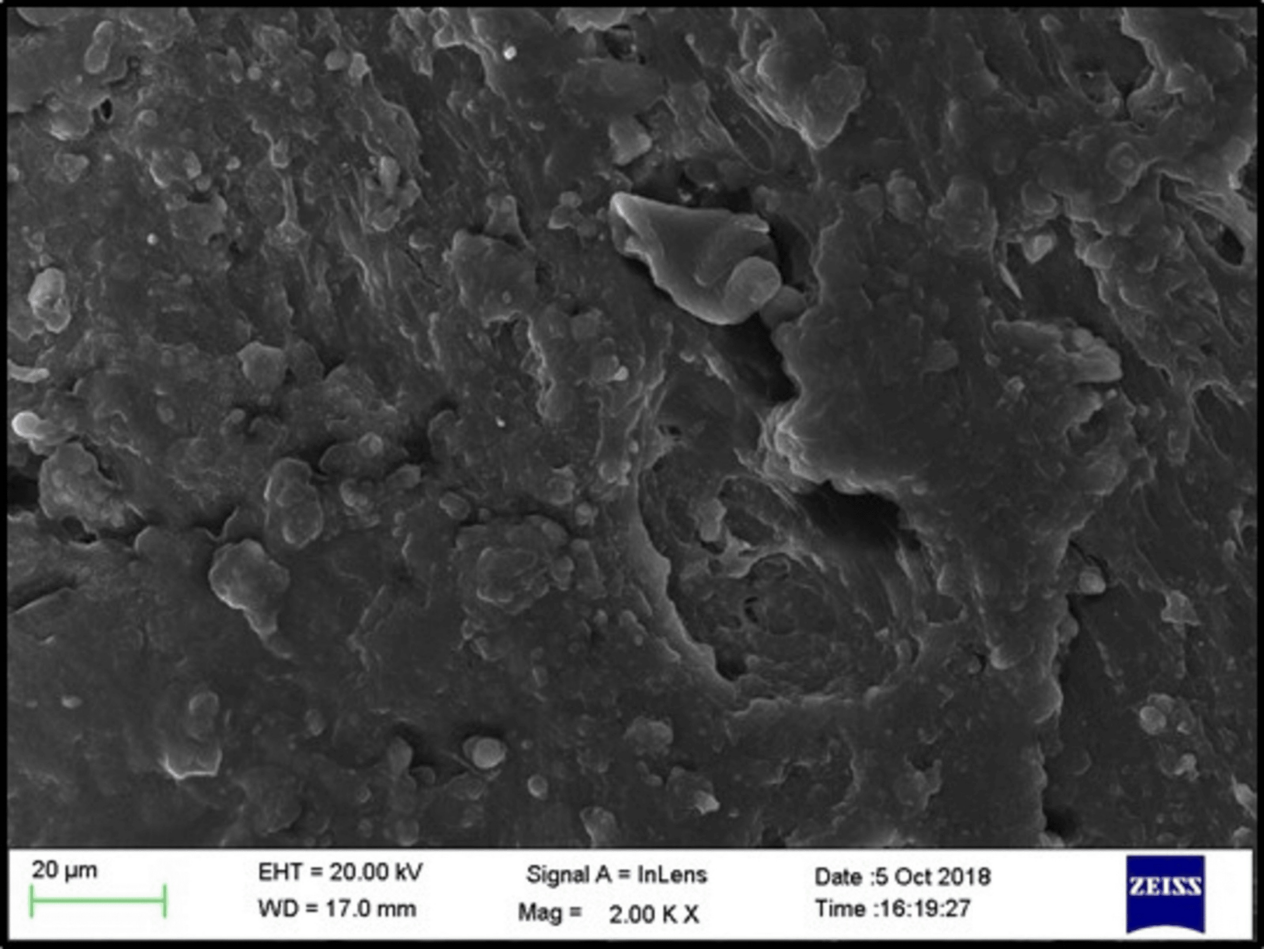
SEM evaluation for sub-group Ep (phosphoric acid) EHT: electron high tension; WD: working distance; SEM: scanning electron microscope

**Figure 5:**
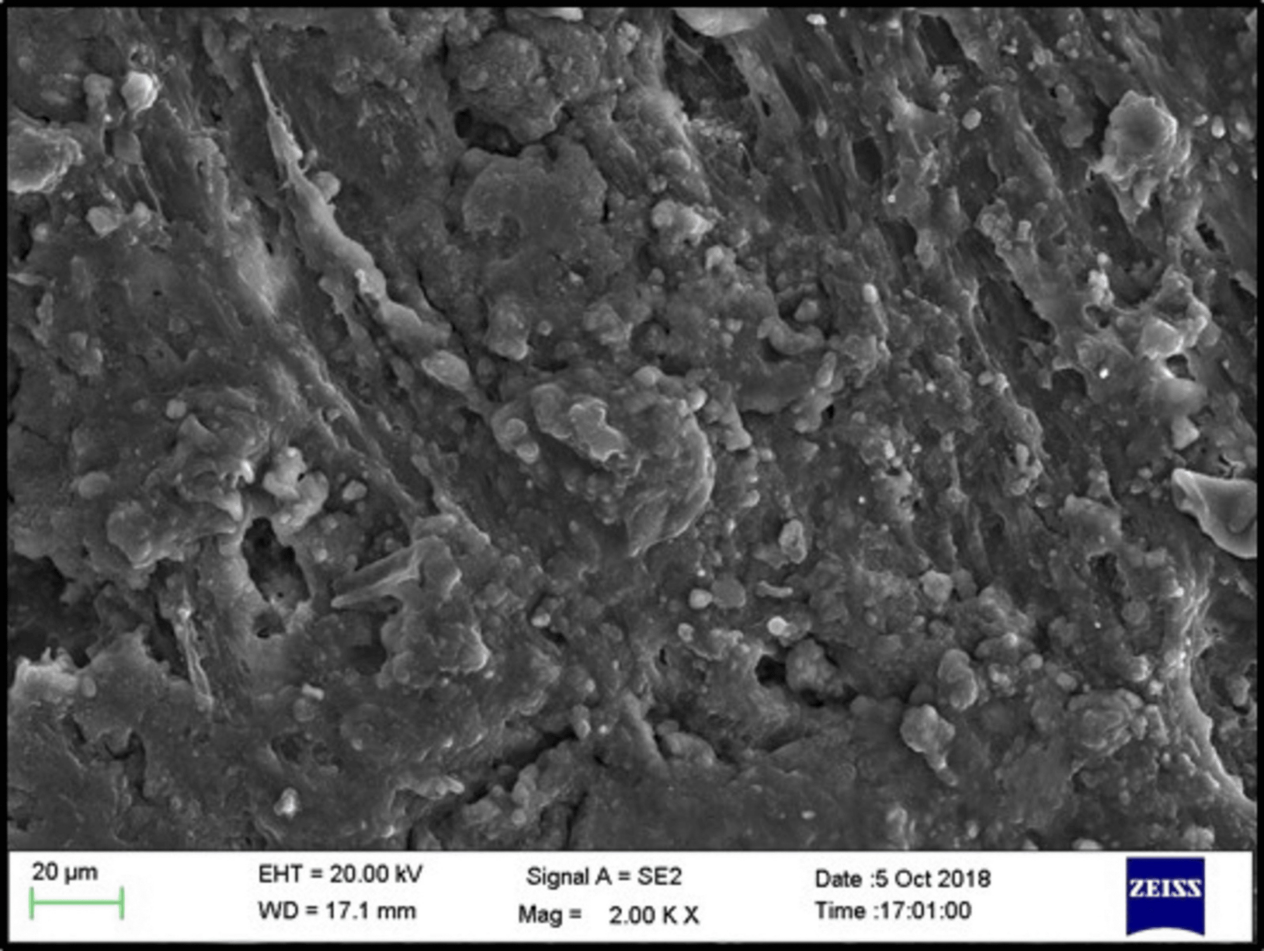
SEM evaluation for sub-group Ea (air abrasion) EHT: electron high tension; WD: working distance; SEM: scanning electron microscope

**Figure 6:**
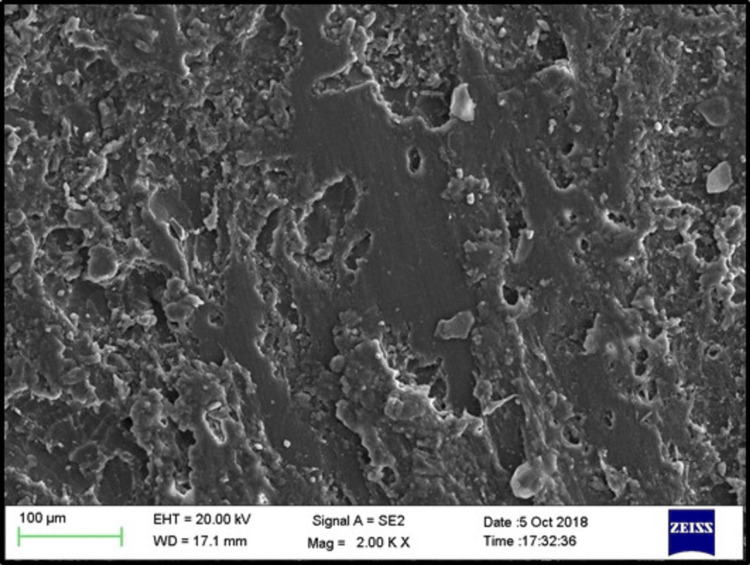
SEM evaluation for sub-group El (Er:YAG laser) EHT: electron high tension; WD: working distance; SEM: scanning electron microscope; Er:YAG: erbium-doped yttrium aluminium garnet

SEM images of the control, phosphoric acid, air abrasion, and laser-treated specimens before bonding revealed that surface treatment created irregularities and small pits on the denture base resin surface (Figures 3-6).

## Discussion

This study compared the flexural strength of heat-polymerized denture base resin after applying different surface treatments and using various repair materials. The results showed significant differences among the groups and subgroups, emphasizing the importance of repair material selection and surface treatment in determining the strength of the repaired denture base.

The findings of this study are consistent with previous research. Alkurt et al. reported that heat-polymerized resin provided the highest flexural strength, whereas autopolymerizing and light-polymerizing resins had lower values [13]. Similarly, Andreopoulos et al. observed that repairs using light-polymerized Triad® Visible Light Cure (VLC) (Dentsply Sirona) resin were weaker than those using autopolymerizing resin [21]. The lower strength of light-polymerized resin may be due to its high viscosity and poor adhesion properties, which make it less effective as a repair material. This could be attributed to the strong cross-linking in heat-polymerized resins, which enhances bonding within the same material. In contrast, light-polymerized resin exhibits limited penetration into polymethylmethacrylate, resulting in weaker adhesion.

While heat-polymerizing resins are generally reported to have superior strength, some studies indicate that light-polymerized acrylic resins can also achieve favorable outcomes. Ogle et al. found that light-polymerized acrylic resins had a more accurate fit and higher strength than heat-polymerized ones [8]. Likewise, Lin et al. reported that VLC resin repairs were stronger than autopolymerizing resin repairs [22]. Similarly, Razavi et al. [23] and Ishigami et al. [24] found no significant differences in the transverse strength of visible light-cured and heat-cured resins. Bural et al. also reported that VLC resin repairs exhibited better mechanical performance than autopolymerizing resin repairs [7].

This study also confirmed that surface treatment significantly improves the flexural strength of repaired heat-polymerized denture base resin. The findings align with those of Minami et al., who found that specimens with surface treatments had higher flexural strength than untreated specimens [25]. Among the different surface treatments, air abrasion and laser treatment were the most effective in improving strength, which aligns with the study by Alkurt et al. [13]. Shimizu et al. also found that air abrasion using 50 µm alumina particles, followed by the application of dichloromethane, improved bond strength when autopolymerizing resin was used for repair [26]. The increased flexural strength observed in this study could be due to improved adhesion resulting from monomer infiltration into micro-pits and surface irregularities, enhancing mechanical interlocking.

SEM analysis revealed that specimens treated with phosphoric acid, air abrasion, and Er:YAG laser exhibited pronounced surface irregularities, such as micro-pits and scratches, which were absent in the untreated control group. These observations align with the findings of Gundogdu et al. [27] and Alkurt et al. [13], who reported that such surface changes enhance mechanical retention by increasing the surface area available for bonding.

Limitations

Despite its valuable findings, this study has some limitations. The lack of artificial aging methods, such as thermocycling and cyclic loading, prevents an assessment of the long-term durability of the repairs. Future research should focus on evaluating the long-term clinical performance of repaired dentures and assessing the impact of various surface treatments on their durability over extended periods.

## Conclusions

The highest flexural strength was observed in specimens repaired with heat-polymerizing resin, regardless of whether surface treatment was applied. Autopolymerizing resin ranked second, followed by light-polymerizing resin. Laser treatment before repairing with heat-polymerizing resin proved to be the most effective method among the surface treatments. Air abrasion was identified as the most effective technique for repairing heat-polymerized denture base resin with autopolymerizing resin. 
